# ﻿Revision of the Chinese endemic genus *Megadrypta* Sciaky & Anichtchenko, 2020 (Coleoptera, Carabidae, Dryptini), with description of a new species

**DOI:** 10.3897/zookeys.1226.142678

**Published:** 2025-02-07

**Authors:** Jia-Heng Chen, Hong-Liang Shi, Hong-Bin Liang

**Affiliations:** 1 College of Forestry, Beijing Forestry University, Beijing, 100083, China Beijing Forestry University Beijing China; 2 Key Laboratory of Zoological Systematics and Evolution, Institute of Zoology, Chinese Academy of Sciences, Beijing 100101, China Chinese Academy of Sciences Beijing China

**Keywords:** China, Dryptinae, ground beetles, new records, new species, taxonomy

## Abstract

The species of the genus *Megadrypta* Sciaky & Anichtchenko, 2020 are reviewed. A new species *M.maozhoui***sp. nov.** is described from Sichuan and Yunnan provinces, China (type locality: Tiantaishan, Qionglai City, Sichuan). New provincial records of *M.mirabilis* Sciaky & Anichtchenko, 2020 are reported as well. A key of the genus *Megadrypta* is provided.

## ﻿Introduction

The Chinese endemic genus *Megadrypta* Sciaky & Anichtchenko, 2020 belonging to the tribe Dryptini was established by Sciaky and Anichtchenko (2020) based on *Megadryptamirabilis* Sciaky & Anichtchenko, 2020 from the Nanling Area in China (north Guangdong and Guangxi provinces).

It comes as a complete surprise to us that there might be additional undiscovered *Megadrypta* species when our dear friends presented us with a large, flightless dryptine specimen collected from Sichuan Province in the summer of 2024. After carefully examining more adult specimens from Sichuan and comparing them with *M.mirabilis*, we can confirm that these specimens represent a new species. Furthermore, examination of specimens from Guizhou and Hunan provinces revealed new provincial distribution records for *M.mirabilis*.

The purposes of this paper are to revise the genus *Megadrypta*, to describe *M.maozhoui* sp. nov., to report new provincial records for *M.mirabilis*, and to present a key to identification of the species of the genus.

## ﻿Materials and methods

Specimens examined in this study were borrowed from or examined in the following institutions and private collections:

**CAU**Entomological Museum, China Agricultural University, Beijing, China

**CBFU**"Collection of Forest Entomology Laboratory, Beijing Forestry University, Beijing, China

**CCJH** personal collection of Jia-Heng Chen, Guangdong, China

**CHYZ** personal collection of Yu-Zhou Huang, Hunan, China

**CYXH** personal collection of Xiao-Han Ye, Zhejiang, China

**IZAS**Institute of Zoology, Chinese Academy of Sciences, Beijing, China

Labels of type material are cited verbatim. Detail information of each label is enclosed within quotation marks (“ ”). Individual labels are separated by double slashes (//). Our added notes are within brackets ([]). Each specimen of the newly described species is provided with one printed label “HOLOTYPE [or] PARATYPE taxon name, det. [author(s)], 2024”. Both holotype and paratype labels are in red.

Most morphological terms in the present paper follow their general applications (Habu 1967; Shi and Liang 2023). The morphological term “apical lamella” is employed to describe the apical portion of median lobe of aedeagus between the extreme apex and the apical orifice, even though this structure is not invariably laminar.

Measurements and abbreviations are as follows : the length of the pronotum (**PL**) was measured along the median line ; the width of the pronotum (**PW**) was measured at the maximum width of the pronotum ; the length of the elytra (**EL**) was measured from the basal border to the sutural apex ; the width of the elytra (**EW**) was measured at the maximum width of the two closed elytra ; the length of the body (**BL**) was the linear distance from the apex of the labrum to the elytral sutural end ; the length of the apical lamella of the aedeagus (**ALL**) was measured from apical margin of apical orifice to the tip of apical lamella ; the width of apical lamella of the aedeagus (**ALW**) was measured along the base of the apical lamella.

The habitus photographs were taken using a Nikon D7100 camera with a Laowa CF 60 mm f/2.8 2× Super Macro lens. Images of external characters and male genitalia characters were taken with a Nikon SMZ-18 stereoscopic dissecting microscope fitted with a Nikon D7500 camera. For each final image, several photographs were taken at different focal planes and combined with Zerene Stacker to get one synthesized photograph. The distribution map was made with QGIS v. 3.28. All images were finally modified and arranged into plates by Adobe Photoshop CC 2019.

## ﻿Results

### ﻿Genus Megadrypta

Taxon classificationAnimaliaColeopteraCarabidae

Sciaky & Anichtchenko, 2020

44ABD71D-6391-5D5D-AE7E-651BF4454556

Megadrypta Sciaky & Anichtchenko, 2020: 525.

#### Type species.

*Megadryptamirabilis* Sciaky & Anichtchenko, 2020, by original designation.

#### Diagnostic characters.

Large dryptine ground beetles with BL 13.0–17.0 mm. Dorsal surface dark with more or less bluish metallic luster; mouth parts, antennae, and legs reddish or dark brown. All terminal palpomeres securiform in males, slenderer and weakly expanded at apex in females; labrum trilobate, with medial lobe forwardly projecting; antennae long, aligned backward reaching middle of elytra; antennomere 1 nearly as long as sum of antennomeres 2–7 combined; temporae well developed. Pronotum cylindrical; anterior angles obtuse; lateral margins of pronotum distinctly doubly concave, before middle and after middle; lateral margins of pronotum completely beaded; posterior angles nearly rectangular, apex rounded. Elytra wide; shoulders extremely narrow; with 6–8 parascutellar pores; striae deeply punctate; intervals slightly convex and pubescent; posterior margins of elytra truncated, slightly sinuate near outer-apical angles. Hind wings reduced. Legs slender, tarsal claws toothless. Male genitalia stout; apical lamella short and wide, apex rounded and thickened; endophallus with more or less distinct central sclerite. Gonocoxite 2 of ovipositor falciform and smooth, without ensiform setae.

#### Geographical notes

**Figure 1.**
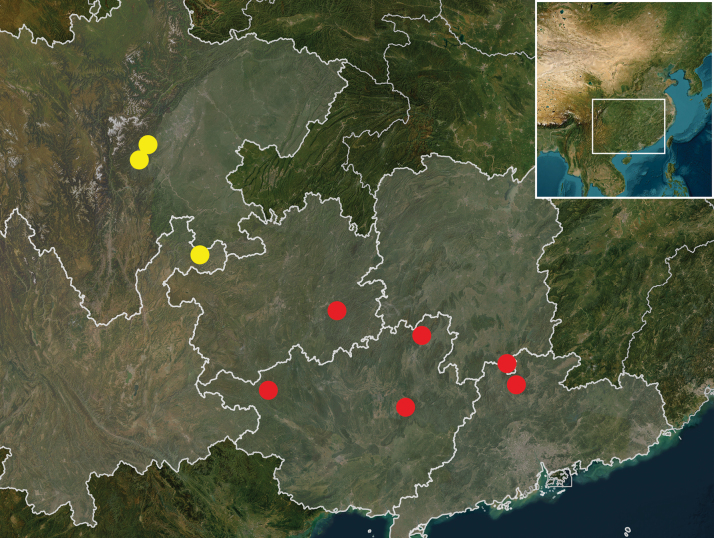
Distribution map for *Megadrypta* spp. Red circles: *M.mirabilis* Sciaky & Anichtchenko; yellow circles: *M.maozhoui* sp. nov.

(Fig. 1). Members of this genus were only found in mid-elevation (1000–1500 m) habitats in southern China. *Megadryptamirabilis* is distributed in the Nanling Area including southeastern Guizhou, northern Guangxi, and northern Guangdong. *Megadryptamaozhoui* sp. nov. is distributed in southwestern mountains around the Sichuan Basin, and it is currently recorded from central Sichuan and northeastern Yunnan.

##### ﻿Key to species of *Megadrypta* Sciaky & Anichtchenko, 2020

**Table:** 

1	Body very large (BL 13–17 mm); pronotal lateral bead complete; elytron with 6–8 parascutellar pores; elytra strongly narrowed toward shoulders; hind wings strongly reduced; gonocoxite 2 of ovipositor without setae	**2 (*Megadrypta*)**
–	Body smaller in size (BL < 14 mm); pronotal lateral bead complete or not; elytron with 1–4 parascutellar pores; elytra less pronouncedly narrowed toward shoulders; hind wings well developed; gonocoxite 2 of ovipositor setose (except for the genus *Nesiodrypta* Jeannel, 1949 without setae)	**other Dryptini genera**
2	Dorsal surface black, with bluish luster very faint and hardly visible; femora dark brown to piceous, tibiae and tarsi sometimes lighter (Fig. 2B); mentum with anterior margin straight at middle (Fig. 6B); elytral striae incised with large punctures (much smaller than interval width); in lateral view, median lobe of aedeagus equally broadened before apical third and then abruptly narrowed, ventral margin strongly curved before apical lamella (Fig. 4A)	***M* . *mirabilis* Sciaky & Anichtchenko**
–	Dorsal surface dark with distinct bluish metallic luster; legs entirely reddish brown (Fig. 2A); mentum with anterior margin concave at middle (Fig. 6A); elytral striae interrupted by very coarse foveae (of similar size as interval width) (Fig. 3C); in lateral view, median lobe of aedeagus gradually attenuate to apex, ventral margin almost straight before apical lamella (Fig. 5A)	***M* . *maozhoui* sp. nov.**

### ﻿ Megadrypta maozhoui sp. nov.

Taxon classificationAnimaliaColeopteraCarabidae

BBE47804-2F23-5671-96C8-BC6D11FFE30A

https://zoobank.org/A513A8DB-BB5D-46C8-9407-8C0DA236DC10

**Figure 2.**
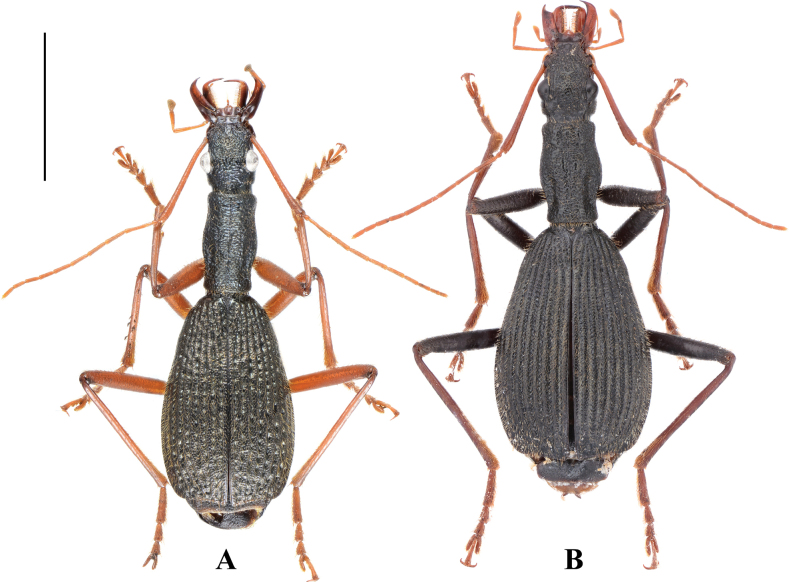
Habitus of *Megadrypta* spp. **A** holotype of *M.maozhoui* sp. nov. **B** female of *M.mirabilis* from Hunan. Scale bar: 5.0 mm.

**Figure 3.**
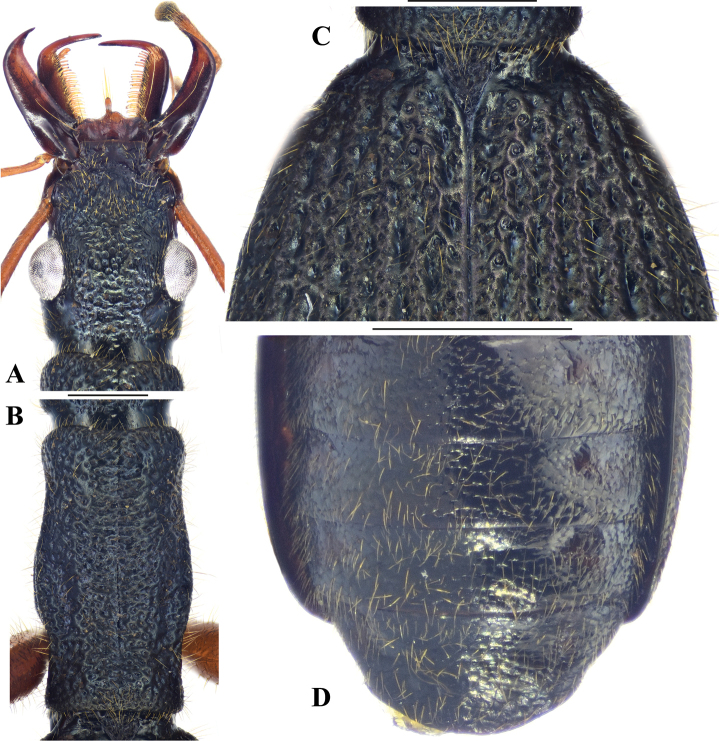
Holotype of *Megadryptamaozhoui* sp. nov. **A** head in dorsal view **B** pronotum **C** basal elytra **D** abdomen. Scale bars: 1.0 mm (**A–C**); 2.0 mm (**D**).

Figs 2A , 3 , 5 , 6A , 7 Chinese vernacular name: 茂洲巨逮步甲

#### Type materials.

***Holotype*: China** • male, labeled “Sichuan, Qionglai, Mt. Tiantaishan, Sandaowan, 1246 m, 2024.V.1-5, N30.2674 E103.1199, Maozhou XU & Tianxuan GU leg.”; “HOLOTYPE *Megadryptamaozhoui* sp. nov., det. Chen & Shi. 2024” [red label]; IZAS. ***Paratypes*: China** • 2 male and 1 female, labeled “Sichuan, Qionglai, Mt. Tiantaishan, Sandaowan, 1246 m, 2024.V.30, N30.2674 E103.1199, Maozhou XU leg.”; CCJH. **China** • 1 female, labeled “Yunnan, Zhaotong, Yiliang County, Luowang Township, Aiziping, N27.8809 E104.6809, 1311 m, 2024.9.9, Anxian SHI leg.”; CAU. **China** • 1 female, labeled “Sichuan, Ya’an, Mt. Zhougongshan, 2018.7, Yong WANG leg.” [alcohol immersion]; IZAS.

#### Diagnosis.

Dorsal surface dark with distinct bluish metallic luster, mouth parts, antennae and all parts of legs reddish brown. Mentum with anterior margin concave and shallowly emarginate at middle. Pronotum with lateral margins gradually sinuate twice, behind anterior angles and before posterior angles. Elytra with 7–8 parascutellar pores, significantly narrowed base and discontinuous striae, interrupted by coarse foveae (of similar size as interval width) (Fig. 3C). Apical lamella of median lobe of aedeagus short (ALL/ALW 0.55–0.61), obtuse and rounded (Fig. 5B, C); median lobe of aedeagus in lateral view stout, gradually attenuate to apex, ventral margin very faintly curved before apical lamella (Fig. 5A, D); endophallus with a distinct central sclerite (Fig. 5C, D).

#### Comparisons.

*Megadryptamaozhoui* sp. nov. can be easily distinguished from the type species of the genus by the combination of following characters: (1) dorsum with distinct bluish metallic luster (versus dark without or with very faint metallic luster in *M.mirabilis*); (2) legs entirely reddish brown, always much paler than that of the latter species; (3) mentum with anterior margin concaved and shallowly emarginate at middle (versus straight and without emargination in *M.mirabilis*); (4) pronotum narrower PL/PW 1.76–1.88 (versus PL/PW 1.62–1.68 in *M.mirabilis*); (5) elytra more elongated, EL/EW 1.56–1.68 (versus EL/EW 1.44–1.50 in *M.mirabilis*); (6) elytral striae discontinuous, interrupted by coarse foveae (versus striae entire with normal punctures in *M.mirabilis*); (7) median lobe of aedeagus in lateral view gradually attenuate to apex, ventral margin nearly straight before apex (versus median lobe evenly thickened before apical third and then abruptly narrowed, ventral margin distinctly curved before apex in *M.mirabilis*); (8) apical lamella of aedeagus with apex obtuse and rounded in dorsal view (versus a little narrower in the latter species); (9) central sclerite of endophallus more distinctly sclerotized with clear border in ventral view (versus less sclerotized with ambiguous border in the latter species).

#### Description.

**Figure 4.**
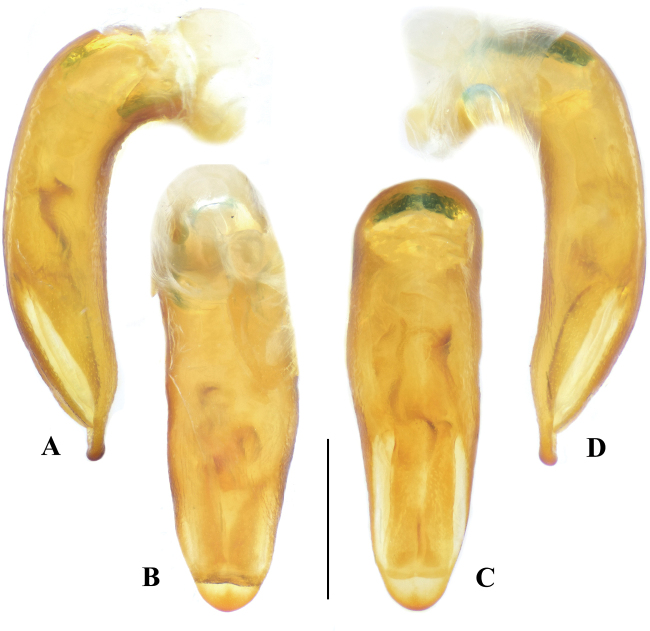
Median lobe of aedeagus, *Megadryptamirabilis*. **A** right lateral view **B** ventral view **C** dorsal view **D** left lateral view. Scale bar: 1.0 mm.

**Figure 5.**
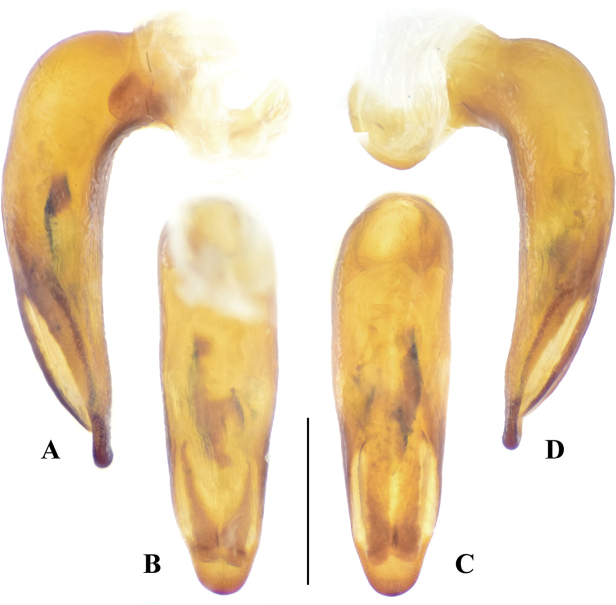
Median lobe of aedeagus, a paratype of *Megadryptamaozhoui* sp. nov. from Tiantaishan, Sichuan **A** right lateral view **B** ventral view **C** dorsal view **D** left lateral view. Scale bar: 1.0 mm.

BL 13.6–15.5 mm (13.8 mm in holotype); dorsal and ventral surface with distinct bluish metallic luster; palpi, antennae and all parts of legs reddish brown, mandibles dark brown. ***Head*** (Fig. 3A): mandibles long, about five times as long as labrum, hooked at apex; labrum trilobate, medial lobe slightly extended anteriorly, anterolateral angles rounded-obtuse, sparsely pubescent near anterior margin; clypeus impunctate and pubescent; vertex densely and coarsely punctate; temporae slightly shorter than eyes, well tumid behind eyes, superficially pubescent and punctate; antennae long, a little exceeding midpoint of elytra. ***Pronotum*** (Fig. 3B) cylindrical (PL/PW 1.76–1.88), disc pubescent and coarsely punctate; anterior angles obtuse; lateral margins distinctly doubly sinuate behind anterior angles and before posterior angles; lateral margins entirely beaded; posterior angles nearly rectangular, apex rounded; median line shallow but distinct; sides of pronotum flattened from above and a little laterally extended near middle, median region elevated. ***Elytra*** (Fig. 3C) wide (EL/EW 1.56–1.68), widest on apical third; with 7–8 parascutellar pores; base strongly narrowed; intervals slightly convex and evenly pubescent; striae discontinuous, interrupted by coarse foveae, the foveae much wider than parascutellar pores, in similar size of interval width; posterior margins truncated, slightly sinuate near outer-apical angles; outer-apical angles obtuse, sutural angles acute angled. ***Male genitalia*** (Fig. 5): median lobe of aedeagus relatively stout, gradually attenuate to apex; in lateral view, median lobe widest near base, ventral margin very weakly curved before apical lamella (Fig. 5A–D), apical lamella slightly thickened apically; median lobe in dorsal view (Fig. 4B, C), with short (ALL/ALW 0.55–0.61), obtuse and rounded apical lamella; endophallus with a distinct central sclerite, the central sclerite axe-shaped, with well-defined border in ventral view.

**Figure 6.**
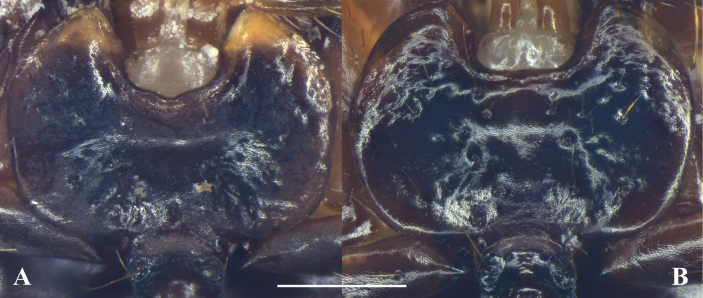
Mentum of *Megadrypta* spp. **A***M.maozhoui* sp. nov. **B***M.mirabilis*. Scale bar: 0.5 mm.

**Figure 7.**
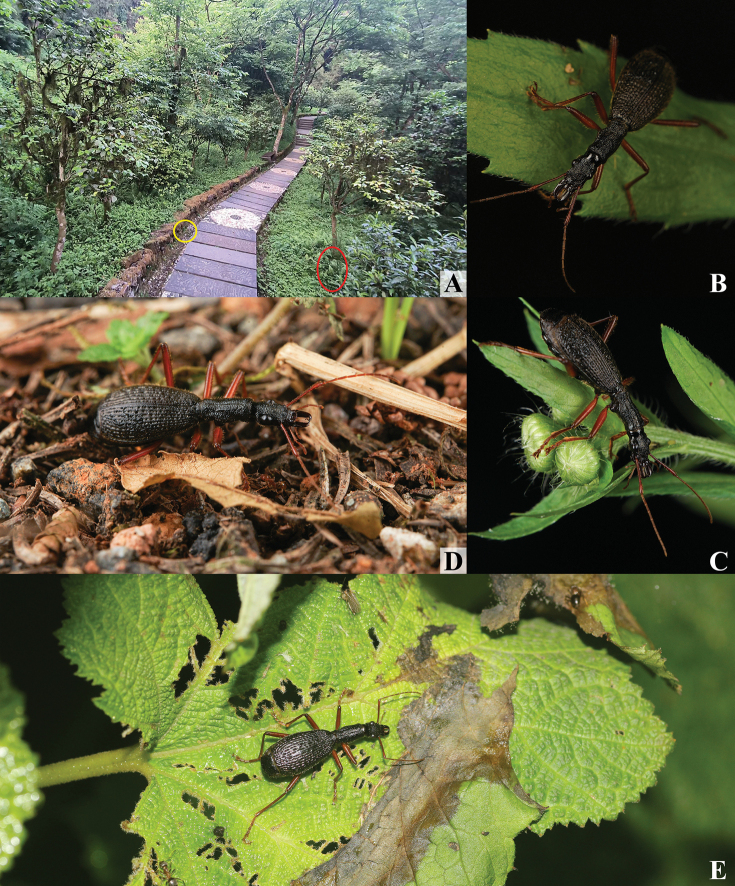
Collecton site and habitus of *M.maozhoui* sp. nov. **A** Collection site on Mount Tiantaishan, Sichuan (**B** and **C** photographed in area of the red circle, **D** photographed in area of yellow circle) **B–D** habitus (photographed by Mao-Zhou Xu) **E** habitus, from Mount Zhougongshan (photographed by Yong Wang).

#### Etymology.

This species is named after Mr Mao-Zhou Xu, who collected and donated most of the type specimens.

#### Distribution.

This species is known from three localities in two provinces of southwestern China: Mount Tiantaishan and Mount Zhougongshan of central Sichuan, and Luowang Township of northeastern Yunnan.

#### Habitat.

(Fig. 7) All specimens were collected on low vegetation under latifoliate forest at night.

### ﻿ Megadrypta mirabilis

Taxon classificationAnimaliaColeopteraCarabidae

Sciaky & Anichtchenko, 2020

06017AE7-6FCB-5E6A-856E-C278A60E4487

Figs 2B , 4 , 6B Chinese vernacular name: 巨逮步甲

Megadrypta mirabilis Sciaky & Anichtchenko, 2020: 526.

#### Material examined.

***Holotype***: China • male, labeled “CHINA. Guangdong, Ruyuan, Nanling Nature Reserve, 24.92579, 113.01638”; “1021 m, 2007.6.18–21 Huang Hao”; IZAS. ***Paratype***: China • 1 male, labeled “CHINA. Guangxi Prov., Cenwanglao Shan Nature Reserve”; “2007.8.5 day, Liu Chunxiang coll.”; IZAS.

#### Non-type materials.

China • 1 female, labeled “CHINA: Hunan, Mt. Mangshan, Jiangjunzhai [in Chinese], 2020.VII-28, 1200 m, Leg. Yuzhou Huang”; CHYZ • 1 female, labeled “CHINA: Guangxi, Laibin, Jinxiu County, Changtong Township, Guitian Village, 2024.VI-1, leg. Chunfu FENG”; CCJH • 1 female, labeled “Guangdong, Nanling, 2017-8” [in Chinese]; CCJH • 1 male, labeled “CHINA: Guizhou, Leishan County, Mt. Leigongshan, Xiannütang, 1555 m” [in Chinese]; “2024.V.31, 26.37303146N 108.19808469E, Leg. Ye”; CYXH.

#### Diagnosis.

Dorsal surface nearly black, with very weak bluish luster; femora dark brown to piceous; mouth parts, tibiae, and tarsi sometimes lighter. Mentum with anterior margin straight at middle. Elytra narrowed toward shoulders; elytral striae incised with large punctures (much smaller than interval width). Median lobe of aedeagus in lateral view equally broadened before apical third and then abruptly narrowed; ventral margin strongly curved before apex.

#### Supplementary description.

BL 13.9–16.1 mm (15.0 mm in the holotype); dorsal and ventral surface nearly black, with very weak bluish luster; mouth parts brown; femora dark brown to piceous; tibiae and tarsi sometimes lighter (Fig. 2B). ***Head***: labrum trilobate; medial lobe slightly extended anteriorly; anterolateral angles rounded-obtuse, sparsely pubescent near anterior margin; clypeus impunctate and pubescent; vertex densely and coarsely punctate; temporae slightly shorter than eyes, well tumid behind eyes, sparsely and finely pubescent and punctate. ***Pronotum***: PL/PW 1.62–1.68; lateral margins gradually sinuate twice, behind anterior angles and before posterior angles; sides flattened from above and a little extended laterally near middle, median region elevated. ***Elytra***: EL/EW 1.44–1.50; base with 7–8 parascutellar pores, strongly narrowed at shoulders; elytral striae incised with large punctures (much smaller than interval width). ***Male genitalia***: median lobe of aedeagus relatively stout (Fig. 4), equally thickened before apical third and then abruptly narrowed in lateral view, with ventral margin strongly curved before apical lamella (Fig. 4A, D); same in dorsal view, with apical lamella short (ALL/ALW 0.46–0.51), obtuse and rounded; endophallus with a central sclerite, hook-shaped in right lateral view (Fig. 4A), with blurred border in ventral view (Fig. 4B).

#### Distribution.

This species is widely distributed in the Nanling Area of southern China (Fig. 1), where it is currently recorded from four provinces: in southern Guizhou from Mount Leigongshan (new provincial record); in southern Hunan from Mount Mangshan (new provincial record); in north Guangxi from Mounts Maoershan, Dayaoshan, and Cenwanglaoshan; and in northern Guangdong from the Nanling Natural Reserve.

## Supplementary Material

XML Treatment for Megadrypta

XML Treatment for Megadrypta maozhoui

XML Treatment for Megadrypta mirabilis

## ﻿Additional information

### Author ORCIDs

Jia-Heng Chen https://orcid.org/0000-0003-2449-8838

Hong-Liang Shi https://orcid.org/0000-0002-9989-5830

Hong-Bin Liang https://orcid.org/0000-0002-9668-1167

### Data availability

All of the data that support the findings of this study are available in the main text.
